# Association between Pneumonia Development and Virulence Gene Expression in Carbapenem-Resistant *Acinetobacter baumannii* Isolated from Clinical Specimens

**DOI:** 10.1155/2023/8265683

**Published:** 2023-12-21

**Authors:** Ji Yun Bae, Ina Yun, Kang Il Jun, Chung-Jong Kim, Miae Lee, Hee Jung Choi

**Affiliations:** ^1^Department of Internal Medicine, Ewha Womans University Mokdong Hospital, Seoul, Republic of Korea; ^2^Ewha Education and Research Center for Infection, Seoul, Republic of Korea; ^3^Department of Internal Medicine, Ewha Womans University Seoul Hospital, Seoul, Republic of Korea; ^4^Department of Laboratory Medicine, Ewha Womans University Mokdong Hospital, Seoul, Republic of Korea; ^5^Department of Laboratory Medicine, Seegene Medical Foundation, Seoul, Republic of Korea

## Abstract

We investigated the virulence gene expression of carbapenem-resistant *Acinetobacter baumanii* (CRAB) isolated from the respiratory samples of patients with CRAB pneumonia and those with CRAB colonization to identify the virulence genes contributing to CRAB pneumonia's development and mortality. Patients with CRAB identified from respiratory specimens were screened at a tertiary university hospital between January 2018 and January 2019. Patients were classified into CRAB pneumonia or CRAB colonization groups according to predefined clinical criteria. *A. baumannii* isolated from respiratory specimens was examined for the expression levels of *ompA*, *uspA*, *hfq*, *hisF*, *feoA*, and *bfnL* by quantitative reverse-transcription polymerase chain reaction. Among 156 patients with CRAB from respiratory specimens, 17 and 24 met the criteria for inclusion in the pneumonia and colonization groups, respectively. The expression level of *ompA* was significantly higher in the pneumonia group than in the colonization group (1.45 vs. 0.63, *P*=0.03). The expression levels of *ompA* (1.97 vs. 0.86, *P*=0.02), *hisF* (1.06 vs. 0.10, *P* < 0.01), *uspA* (1.62 vs. 1.01, *P* < 0.01), and *bfnL* (3.14 vs. 2.14, *P*=0.03) were significantly higher in patients with 30-day mortality than in the surviving patients. Elevated expression of *hisF* (adjusted odds ratio = 5.93, *P*=0.03) and *uspA* (adjusted odds ratio = 7.36, *P*=0.02) were associated with 30-day mortality after adjusting for age and the Charlson score. *uspA* and *hisF* may serve as putative targets for novel therapeutic strategies.

## 1. Introduction

*Acinetobacter baumannii* is the leading cause of ventilator-associated pneumonia in patients in intensive care units (ICU); however, it can also cause community-acquired pneumonia [1, 2]. Among *A. baumannii*, carbapenem resistance is increasing in A. baumannii, reaching 50–70% depending on the region [3–6]. Carbapenem-resistant *A. baumannii* (CRAB) has doubled the mortality rate of carbapenem-sensitive *A. baumannii* [7] because of the lack of optimal treatment options. Thus, CRAB is a critical global priority, according to the World Health Organization [8].

Several host factors such as ICU stay, recent surgery, comorbidities, immunosuppressive therapy, and tracheostomy have been suggested as risk factors for *A. baumannii* invasive infections [9, 10]. In addition to host factors, understanding the microbiological factors associated with pneumonia is important because these factors may contribute to developing novel treatment strategies and early diagnostic tools for *A. baumannii* pneumonia [11].

Several virulence mechanisms contribute to *A. baumannii* lung infections, including biofilm production, attachment to biotic surfaces, host cell invasion, apoptosis, oxidative stress resistance, and iron regulation [8]. Several virulence genes have been reported to contribute to this process. *ompA* encoding the outer membrane protein (OmpA) enhances cell death via mitochondrial and nuclear targeting [12–15]. Universal stress protein A (UspA) is responsible for resistance to oxidative stress and low pH [16]. The RNA chaperone Hfq contributes to resistance to stresses such as temperature, pH, osmotic pressure, oxidative stress, adhesion, and invasion of host cells and modulates fimbriae production [17]. HisF decreases innate immunity and the inflammatory responses [18]. FeoA plays roles in cell fitness, adhesion, biofilm formation, and growth [19]. *bfnL*, which belongs to the baumanoferrin cluster, is involved in biofilm formation, attachment, and fitness under iron-limiting condition [14]. The deletion of these genes leads to a loss of virulence, as mice infected with the knockout strain showed improved survival [14, 16, 18, 20]. The elevated expression of *ompA*, *feoA*, *bfnL*, and *hisF* has been observed in murine pneumonia infections [14]. Because this knowledge is based on in vitro experiments and animal studies, it is necessary to investigate the virulence gene expression of *A. baumannii* in human infections.

This study aimed to investigate the virulence gene expression of CRAB isolated from respiratory samples of patients with CRAB pneumonia and those with CRAB colonization and to demonstrate the virulence genes contributing to the development of pneumonia and subsequent mortality.

## 2. Materials and Methods

### 2.1. Study Design

The study subjects were inpatients at a tertiary university hospital between January 2018 and January 2019, for whom CRAB was identified from respiratory specimens. The inpatients included patients admitted to the ICU. Respiratory specimens included sputum, endotracheal aspirates, and bronchoalveolar lavage fluid. Respiratory cultures were performed according to the order of the attending physician as part of usual clinical practice. The expectorated sputum specimens submitted for culture were evaluated for their quality using the modified Murray-Washington grouping system [21]. Only group 4–6 specimens were considered acceptable and proceeded for culture. Transtracheal aspirate through an endotracheal tube or bronchial aspirate through bronchoscopy were considered as lower respiratory tract specimens, regardless of the squamous epithelial cell count. The respiratory specimens were inoculated on the blood agar plate (BAP) and the MacConkey agar and incubated in 5% CO_2_ at 36°C for 1-2 days. *A. baumannii* was identified using VITEK MS (bioMérieux, France). The carbapenem resistance of *A. baumannii* was tested using VITEK2 (bioMérieux, France) with an AST-N225 card (bioMérieux, France). A minimum inhibitory concentration (MIC) of ≥8 *µ*g/mL for imipenem and meropenem was considered to indicate carbapenem resistance, according to the Clinical and Laboratory Standards Institute guidelines [22]. The isolated CRAB was stored at −80°C until further analysis.

The screened patients were retrospectively determined to have CRAB pneumonia if they met the following criteria: (a) radiologic evidence of pneumonia at the time of CRAB isolation, (b) ≥5 days of effective antibiotic treatment initiated within three days of identification of *A. baumannii*, and (c) no other microorganisms identified from blood or sputum within one month of isolation of *A. baumannii.* In criterion (b), five days represent the minimal duration needed to treat pneumonia, and three days represent a reasonable interval to consider that the antibiotic prescription was intended to treat the isolates. The criterion (c) was used to exclude polymicrobial infections. Additionally, patients who satisfied criteria (a) and (c) but died before receiving five or more days of effective antibiotic treatment, with pneumonia as the primary cause of death, were considered to have CRAB pneumonia.

Screened patients were determined to have CRAB colonization if they met (d) no radiologic evidence of pneumonia within seven days of the isolation of *A. baumannii* and (e) a lack of coexistence of *A. baumannii* bacteremia.

CRAB isolated from patients with pneumonia and colonization was used for further molecular analysis. If patients met the above criteria for more than one CRAB isolate, only the first isolate was included in the analysis.

### 2.2. Molecular Studies

*A. baumannii* was grown to the late exponential phase in tryptic soy broth (TSB) at 37°C. Bacterial RNA was isolated using an AccuPrep® Bacterial RNA Extraction Kit (Bioneer, Daejeon, Korea). cDNA was obtained following the Transcriptor First-Strand cDNA Synthesis Kit protocol (Roche, Basel, Switzerland) using a T-100 thermal cycler (Bio-Rad Laboratories, Hercules, California, USA). Real-time PCR for *ompA*, *uspA*, *hfq*, *hisF*, *feoA*, and *bfnL* was performed using a LightCycler®96 (Roche, Basel, Switzerland) following the protocol of the FastStart Essential DNA Green Master (Roche, Basel, Switzerland). PCR cycle conditions were 10 s at 95°C, 5 s at annealing temperature, and 10 s at 72°C. Annealing temperatures were 57°C for *feoA*, *bfnL*, and *uspA*, 60°C for *hfq*, 58°C for *ompA*, and 55°C for *hisF*. The primer sequences of each gene were as follows: *ompA* forward: 5′-CGCTTCTGCTGGTGCTGAAT-3′,*ompA* reverse: 5′-CGTGCAGTAGCGTTAGGGTA-3′,*uspA* forward: 5′-TTCTTTGGCAGCAGCACGAC-3′,*uspA* reverse: 5′-CACCTTCTTCAGCGCATAGGG-3′,*hfq* forward: 5′-GCGTGTTTGTAAACCATTTGACTTAC-3′,*hfq* reverse: 5′-CATCCCAGTTTCTATTTTCCTTGTTAAC-3′,*hisF* forward: 5′-AATACAGTCTCGGCCATACG-3′,*hisF* reverse: 5′-TCCTCGATATTCGTGATGCG-3′,*feoA* forward: 5′-GGCGACCATCACCAAAGTGAA-3′,*feoA* reverse: 5′-TGGTAATCACTTCCACTCGCGT-3′,*bfnL* forward: 5′-TACACCGCTGGATGCATGAG-3′,*bfnL* reverse: 5′-AATTTCGGCATACCCCACGT-3′, 16S rRNA forward: 5′-GAGGAAGGTGGGGATGACGT-3′, 16S rRNA reverse: 5′-AGGCCCGGGAACGTATTCAC-3′. The primer sequences for *ompA*, *uspA*, *hfq*, *bfnL*,and 16S rRNA were determined based on previous studies [17, 23–25]. Primer sequences for *feoA* and *hisF* were purchased from Bioneer (Daejeon, Korea). The expression level of each gene was presented as the relative gene expression using the 2^−ΔΔ*Ct*^ method [26]. The median *C*_*t*_ values of the three technical replicates were used. The 16S rRNA gene was used as a housekeeping gene to normalize gene expression, and *A. baumannii* American Type Culture Collection (ATCC) 19606 was used as the calibrator.

### 2.3. Variables

Several demographic and clinical variables were investigated, including age, sex, comorbidities (Charlson score), recent surgery, ICU stay, mechanical ventilation, tracheostomy, and 30-day mortality. The 30-day mortality comprised all-cause deaths within 30 days after *A. baumannii* isolation.

### 2.4. Statistical Analysis

The difference in virulence gene expression levels of CRAB between patients with pneumonia and colonization and between deceased and surviving patients was investigated using the Mann-Whitney *U* test. Other risk factors for developing CRAB pneumonia were compared using Fisher's exact and chi-square tests for categorical variables and the Mann–Whitney *U* test for continuous variables.

Multivariate analysis was performed using logistic regression to investigate whether the overexpression of the virulence gene of *A. baumannii* is an independent risk factor for pneumonia and mortality. The outcome variables were *A. baumannii* pneumonia and 30-day mortality rate. The independent variables were the expression level of each virulence gene, age, and the Charlson Comorbidity Index. Differences were considered statistically significant at *P* < 0.05. All statistical analyses were performed using SPSS software (version 25.0; IBM, Armonk, New York, USA).

## 3. Results

A total of 156 patients with CRAB identified from respiratory specimens were screened during the study period. Among these, 68 patients with polymicrobial infections and 47 patients who did not meet the criteria for pneumonia or colonization were excluded from the study. Finally, 17 and 24 patients met the criteria for pneumonia and colonization and were included in the analysis.

*ompA* expression levels were significantly higher in the pneumonia group than in the colonization group (1.45 vs. 0.63, *P*=0.03). The expression levels of *uspA*, *hfq*, *hisF*, *feoA*, and *bfnL* were not significantly different between groups (Table 1, Figure S1). After adjusting for age and the Charlson score, none of the virulence gene expression levels was significantly associated with pneumonia (Table 2).

Nine of the 17 patients in the pneumonia group and one of the twenty-four patients in the colonizer group died within 30 days of CRAB isolation. The cause of death in the pneumonia group was CRAB pneumonia in eight patients and sepsis other than CRAB in one patient. One patient in the colonization group died of pneumonia caused by microorganisms other than CRAB.

The expression levels of *ompA* (1.97 vs. 0.86, *P*=0.02), *hisF* (1.06 vs. 0.10, *P* < 0.01), *uspA* (1.62 vs. 1.01, *P* < 0.01), and *bfnL* (3.14 vs. 2.14, *P*=0.03) were significantly higher in deceased patients than in surviving patients (Table 3, Figure S2). After adjusting for age and the Charlson score, *hisF* (adjusted odds ratio = 5.93, *P*=0.03) and *uspA* (adjusted odds ratio = 7.36, *P*=0.02) remained associated with 30-day in-hospital mortality (Table 4).

## 4. Discussion

We observed higher *ompA* expression in the CRAB pneumonia patients than in those with colonization. In addition, higher *ompA*, *hisF*, *uspA*, and *bfnL* expression in CRAB was observed in patients with 30-day in-hospital mortality than in the surviving patients. The association between virulence gene expression and pneumonia was not statistically significant after adjusting for age and the Charlson score. *hisF* and *uspA* were significantly associated with 30-day mortality after adjusting for age and the Charlson score.

Given that antibiotic-resistant *A. baumannii* poses a considerable threat to health in nosocomial settings, diverse efforts have been made to reduce the burden of CRAB infections in healthcare settings, such as introducing an antimicrobial restriction system to guide the appropriate use of carbapenem [27]. Many studies have attempted to determine the optimal dose, duration, and combination of traditional antibiotics for treating CRAB. Despite these efforts, there is no precise “standard-of-care antibiotic regimen for CRAB infection yet [28–30].

The management of CRAB infections is complicated for several reasons. First, CRAB is mainly recovered from respiratory specimens. Still, it is not always clear whether an isolate is a colonizing organism or whether CRAB represents a true pathogen, leading to uncertainty regarding the need for antibiotic therapy. Second, once *A. baumannii* exhibits carbapenem resistance, it generally acquires resistance to most other antibiotics expected to be active against wild-type *A. baumannii*. With the emergence of multidrug- and pan-drug-resistant *A. baumannii*, there is an urgent need to identify new methods for treating drug-resistant *A. baumannii* infections [15, 31].

Identifying the virulence gene expression of CRAB in patients with pneumonia and colonization is valuable for two reasons. If there is a significant difference in gene expression between the two, it can be used as an indicator to distinguish between pneumonia and colonization. Second, the overexpression of virulence genes during pneumonia suggests they could be targets for novel therapeutic developments [15, 20]. For example, AOA-2 is a synthetic polypeptide designed to interact with OmpA. AOA-2 inhibits the adhesion of *A. baumannii* to biotic and abiotic surfaces and enhances the sensitivity of *A. baumannii* to antibiotics [32–35]. OmpA is also a potential target for developing vaccines and monoclonal antibodies [15]. Except for the relatively well-known *ompA*, investigations on the virulence genes of *A. baumannii* have been limited to in vitro and animal studies. This is the first study to report the expression levels of *hisF*, *uspA*, *bfnL*, *feoA*, and *hfq* in clinical isolates, which may provide fundamental data for selecting novel therapeutic targets.

OmpA is a surface-exposed porin protein with a *β*-barrel structure embedded in the outer membrane [36]. The amino acids of *A. baumannii* OmpA from a variety of clinical isolates are highly conserved (>89%), but are not homologous to the human proteome [37]. This is a favorable feature of OmpA as a potential therapeutic target. OmpA is involved in the adherence to the epithelia [13], translocation into the epithelial cell nucleus [38], and induction of the epithelial cell death [39]. OmpA is also involved in biofilm formation [13] and binds to factor H, allowing *A. baumannii* to develop serum resistance [40]. If sufficient data are accumulated to set a threshold for predicting the virulence of *A. baumannii*, qRT-PCR monitoring of *A. baumannii ompA* may be a promising tool for detecting the development of pneumonia.

*uspA* has been detected in *A. baumannii* as an open reading frame (ORF) on an AbaR resistance island [41, 42]. *In vitro* studies have shown that UspA is involved in resistance to H_2_O_2_. Because generation of reactive oxygen species is essential to the host's innate immune response, resistance to oxidative stress helps *A. baumannii* survive the host defense mechanism, leading to invasive infection. In an animal study, infection with *uspA-*deleted *A. baumannii* resulted in a reduced bacterial load in the lungs and improved the survival of mice, suggesting its role in the lethality of *A. baumannii*. This is consistent with our results in which elevated *uspA* expression was associated with 30-day mortality. The UspA protein sequence is highly conserved among different *A. baumannii* strains, indicating that it may be a plausible therapeutic target [16].

The HisF is involved in histidine and de novo purine biosynthesis. HisF is involved in inhibiting the recruitment of innate immune cells and decreasing the production of the proinflammatory cytokine IL-6. Thus, HisF decreases innate immunity and inflammatory responses, enabling *A. baumannii* to survive host defense mechanisms and cause invasive infections. Overexpression of *hisF* has been established in a mouse model of pneumonia [14, 18]. In a murine pneumonia model, mice infected with the *hisF*-deleted mutant strain showed a lower bacterial burden in the lungs and an improved survival rate, suggesting that HisF could be a therapeutic target to improve the survival of *A. baumannii* infection. This was consistent with our results, in which elevated *hisF* expression was associated with 30-day mortality.

Belonging to the baumanoferrin cluster, *bfnL* encodes an N-acetyltransferase associated with ferric siderophore synthesis [14, 43]. *bfnL* is involved in bacterial attachment and biofilm formation. Biofilm-forming *A. baumannii* is particularly difficult to treat because it is more resistant to antimicrobial agents and the host immune system than planktonic bacteria. This enables *A. baumannii* to persist on biotic surfaces and diverse medical devices [44]. Deletion of *bfnL* resulted in reduced adherence of *A. baumannii* to human alveolar epithelial cells and decreased biofilm formation. Mice infected with the *bfnL* mutant strain showed improved survival in a pneumonia model [14]. We also identified higher expression of *bfnL* in patients with 30-day mortality, although the association was not significant in multivariable analysis.

We observed a higher *ompA* expression in patients with pneumonia, which is consistent with the results of previous clinical and animal studies. The virulence gene expression effect on pneumonia was insignificant after adjusting for age and the Charlson score. Nevertheless, it is difficult to conclude that virulence gene expression is not associated with pneumonia because the sample size in this study was too small to achieve statistical significance in multivariate analysis. Furthermore, when categorizing patients according to mortality, significantly higher expression of *ompA*, *hisF*, *uspA*, and *bfnL* was noted in patients with 30-day mortality than in those who survived. *hisF* and *uspA* remained significantly associated with 30-day mortality after adjusting for age and the Charlson score. The elevated expression of *hisF*, *uspA*, and *bfnL* in patients with 30-day mortality but not in patients with pneumonia raises two hypotheses. First, the possible misclassification of colonization as pneumonia might have attenuated the differences between the groups. Based on the criteria for pneumonia used in this study, identifying pneumonia on chest radiography and prescribing antibiotics against CRAB were based on clinical decisions that can vary depending on the physician. However, mortality is an objective outcome, and the misclassification of deceased and surviving patients is impossible. A large-scale prospective study in which an assigned investigator determines pneumonia according to predefined criteria combining patients' symptoms and laboratory and imaging findings may improve the grouping and reliability of the multivariable analysis. Second, the expression of these genes may be higher in advanced pneumonia than in mild pneumonia. The analysis of gene expression levels depending on the severity of pneumonia may provide supporting evidence for this hypothesis.

This study has several limitations. First, this was a single-center study with a small sample size. Because we excluded polymicrobial infections and patients who did not meet CRAB pneumonia or colonization criteria, the final number of patients analyzed was small. Second, the multivariate analysis included only age and the Charlson score as covariates. The sample size of this study was too small to include many covariates. We did not include ICU stay and mechanical ventilation in the multivariable analysis because it was uncertain whether these were the result of the pneumonia or the predisposing factors for the pneumonia. In addition, multicollinearity was expected between ICU stay and mechanical ventilation. A prospective study design may help clarify this temporal relationship. Third, we adopted the prescription of antibiotics against CRAB as one of the criteria for pneumonia, assuming that the attending physician decided to treat pneumonia based on symptoms, physical examination findings, and laboratory and imaging studies. However, clinical decisions can vary depending on the physician, which may affect consistency in the definition of pneumonia. A prospective study design with a single assigned investigator judging pneumonia or colonization may improve grouping consistency. Fourth, gene expression analysis was performed under controlled conditions (in TSB broth at 37°C), which did not represent the environment of the living tissue. Whether the overexpression of virulence genes is strain-specific or a response to various environmental stressors needs to be determined. Fifth, unlike in animal studies, *A. baumannii* isolates may be obtained from different time points of pneumonia in each patient, which may partially affect gene expression levels.

## 5. Conclusions

The elevated expression of *hisF* and *uspA* in CRAB is associated with 30-day mortality, suggesting that these genes may be putative targets for novel therapeutic strategies. Higher *ompA* expression in the CRAB of patients with pneumonia than in those with colonization and higher *ompA* and *bfnL* expression in CRAB in patients with 30-day in-hospital mortality than in surviving patients necessitate a large-scale prospective study to establish the usefulness of these genes as targets for novel diagnostic and therapeutic strategies.

## Figures and Tables

**Table 1:** Expression levels of *Acinetobacter baumannii* virulence genes and clinical characteristics of patients with pneumonia and colonization.

	Pneumonia (*n* = 17)	Colonization (*n* = 24)	*P*
Age, median (IQR), y	75 (66–81)	68 (59–78)	0.14
Female sex, no. (%)	4 (23.5)	5 (20.8)	1.00
Charlson score, median (IQR)	5 (4–7)	4 (2–7)	0.16
Surgery, no. (%)	3 (17.6)	8 (33.3)	0.31
ICU stay, no. (%)	16 (94.1)	13 (54.2)	0.01
Tracheostomy, no. (%)	3 (17.6)	10 (41.7%)	0.10
Mechanical ventilation, no. (%)	13 (76.5)	6 (25.0)	<0.01
Death, no. (%)	9 (52.9)	1 (4.2)	<0.01
*ompA*, median (IQR)	1.45 (0.88–2.24)	0.63 (0.13–1.39)	0.03
*uspA*, median (IQR)	1.23 (0.75–1.69)	1.02 (0.83–1.23)	0.29
*hfq*, median (IQR)	1.54 (1.34–1.86)	1.26 (1.02–1.83)	0.06
*hisF*, median (IQR)	0.86 (0.10–1.09)	0.12 (0.07–1.02)	0.16
*feoA*, median (IQR)	1.29 (1.10–1.77)	1.30 (0.78–1.67)	0.37
*bfnL*, median (IQR)	2.33 (2.05–3.51)	2.14 (1.72–3.31)	0.21

IQR, interquartile range; ICU, intensive care unit.

**Table 2:** Association of *Acinetobacter baumannii* virulence gene expression levels and pneumonia adjusted for age and Charlson score.

	Adjusted OR (95% CI)	*P*
*ompA*	1.46 (0.83–2.57)	0.19
*uspA*	2.54 (0.55–11.80)	0.23
*hfq*	1.44 (0.66–3.14)	0.36
*hisF*	1.86 (0.56–6.21)	0.31
*feoA*	1.33 (0.48–3.69)	0.59
*bfnL*	1.45 (0.81–2.60)	0.21

OR, odds ratio; CI, confidence interval.

**Table 3:** Expression levels of *Acinetobacter baumannii* virulence genes and clinical characteristics of deceased and surviving patients.

	Deceased (*n* = 10)	Survived (*n* = 31)	*P*
Age, median (IQR), y	77 (71–82)	66 (58–78)	0.10
Female sex, no. (%)	3 (30.0)	6 (19.4)	0.66
Charlson score, median (IQR)	5 (4–7)	4 (2–7)	0.26
ICU stay, no. (%)	9 (90.0)	20 (64.5)	0.23
Mechanical ventilation, no. (%)	6 (60.0)	13 (41.9)	0.47
*ompA*, median (IQR)	1.97 (0.74–2.85)	0.86 (0.15–1.40)	0.02
*hisF*, median (IQR)	1.06 (0.90–1.21)	0.10 (0.08–0.86)	<0.01
*uspA*, median (IQR)	1.62 (1.16–1.75)	1.01 (0.64–1.21)	<0.01
*hfq*, median (IQR)	1.54 (1.39–1.74)	1.31 (1.13–2.00)	0.19
*feoA*, median (IQR)	1.61 (1.09–1.84)	1.20 (0.84–1.66)	0.15
*bfnL*, median (IQR)	3.14 (2.42–3.86)	2.14 (1.75–2.53)	0.03

IQR, interquartile range; ICU, intensive care unit.

**Table 4:** Association of *Acinetobacter baumannii* virulence gene expression and 30 day in-hospital mortality adjusted for age and Charlson score.

	Adjusted OR (95% CI)	*P*
*ompA*	1.83 (0.98–3.43)	0.06
*hisF*	5.93 (1.24–28.25)	0.03
*uspA*	9.02 (1.27–64.19)	0.03
*hfq*	0.92 (0.38–2.21)	0.85
*feoA*	1.66 (0.54–5.09)	0.37
*bfnL*	1.59 (0.86–2.95)	0.14

OR, odds ratio; CI, confidence interval.

## Data Availability

The data presented in this study are principally contained in the manuscript and are available upon reasonable request from the corresponding author.

## Supplementary Materials

Supplementary Materials Supplementary Figure 1: CRAB virulence gene expression in patients with pneumonia and colonization. The relative gene expression was presented using the 2^−ΔΔ*Ct*^ method with the 16S rRNA as a housekeeping gene and ATCC19606 as the calibrator. The horizontal line represents the median value of each group. Supplementary Figure 2: CRAB virulence gene expression in patients with 30-day in-hospital mortality and surviving patients. The relative gene expression was presented using the 2^−ΔΔ*Ct*^ method with the 16S rRNA as a housekeeping gene and ATCC19606 as the calibrator. The horizontal line represents the median value of each group.
