# Supplementary material for: Association between Pneumonia Development and Virulence Gene Expression in Carbapenem-Resistant Acinetobacter baumannii Isolated from Clinical Specimens
Source: Can J Infect Dis Med Microbiol. 2023 Dec 21;2023:8265683. doi: 10.1155/2023/8265683 (PMC10754638; doi:10.1155/2023/8265683)
Supplement: Supplementary Materials — Supplementary Figure 1: CRAB virulence gene expression in patients with pneumonia and colonization. The relative gene expression was presented using the 2−ΔΔCt method with the 16S rRNA as a housekeeping gene and ATCC19606 as the calibrator. The horizontal line represents the median value of each group. Supplementary Figure 2: CRAB virulence gene expression in patients with 30-day in-hospital mortality and surviving patients. The relative gene expression was presented using the 2−ΔΔCt method with the 16S rRNA as a housekeeping gene and ATCC19606 as the calibrator. The horizontal line represents the median value of each group. [file 8265683.f1.docx]

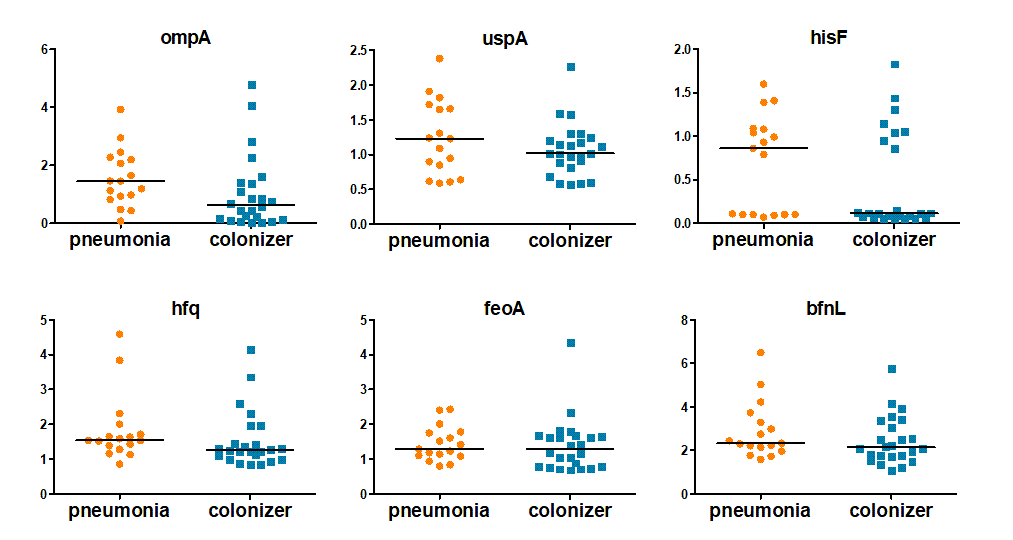


Figure S1. CRAB virulence gene expression in patients with pneumonia and colonization. The relative gene expression was presented using the 2^-ΔΔCt^ method with the 16S ribosomal RNA as a housekeeping gene and ATCC19606 as the calibrator. The horizontal line represents the median value of each group.


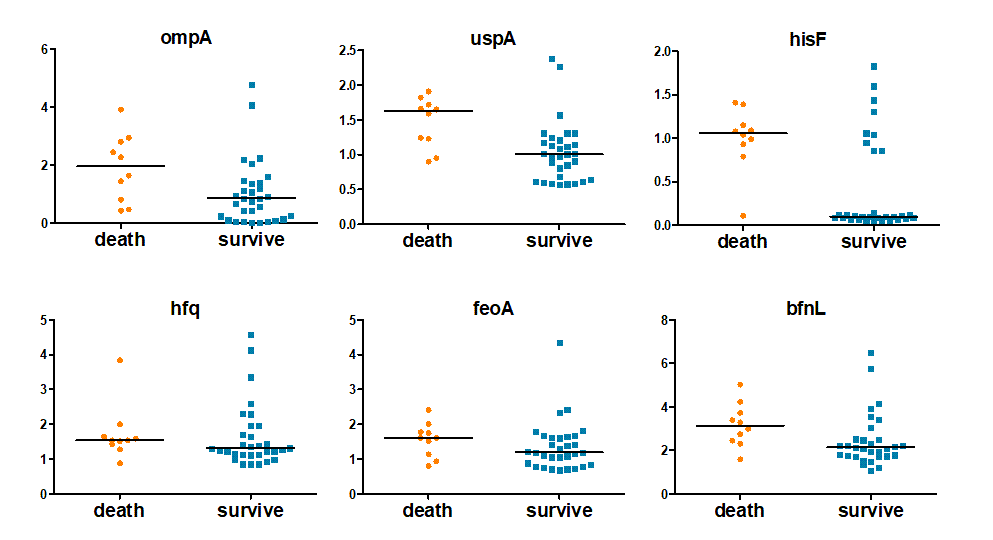


Figure S2. CRAB virulence gene expression in patients with 30-day in-hospital mortality and surviving patients. The relative gene expression was presented using the 2^-ΔΔCt^ method with the 16S ribosomal RNA as a housekeeping gene and ATCC19606 as the calibrator. The horizontal line represents the median Ct value of each group.
